# Tendances des prévalences du VIH et de la syphilis chez les femmes enceintes en consultation prénatale au Togo : Analyse des résultats de la sérosurveillance sentinelle entre 2008 et 2016

**DOI:** 10.48327/mtsi.v2i3.2022.152

**Published:** 2022-07-13

**Authors:** Abla Séfako AKAKPO, Aminou LARABOU, Bayaki SAKA, Julienne Noude TÉCLESSOU, Assetina SINGO, Anoumou DAGNRA, Koussake KOMBATÉ, Palokinam PITCHÉ

**Affiliations:** 1 Service de dermato-vénéréologie, Centre hospitalier universitaire Sylvanus Olympio, Université de Lomé, Togo; 2 Service de dermato-vénéréologie, Centre hospitalier universitaire Campus, Université de Lomé, Togo; 3 Programme national de Lutte contre le Sida et les Infections sexuellement transmissibles, Ministère de la Santé publique, Lomé, Togo; 4 Conseil national de Lutte contre le Sida et les Infections sexuellement transmissibles, Lomé, Togo

**Keywords:** Femmes enceintes, VIH, Syphilis, Hôpital, Lomé, Togo, Afrique subsaharienne, Pregnant women, HIV, Syphilis, Hospital, Lomé, Togo, Sub-Saharan Africa

## Abstract

**Introduction:**

Le but de notre étude était d'analyser les tendances de l'infection à VIH et de la syphilis chez les femmes enceintes en consultation prénatale (CPN) dans les structures de soins du Togo.

**Méthodes:**

Il s'est agi d'une étude rétrospective analytique, couvrant la période de 2008 à 2016 et portant sur les femmes enceintes de 15 à 49 ans vues en CPN pour la première fois dans les services de santé maternelle et infantile au Togo.

**Résultats:**

Durant la période d’étude, 41 536 femmes enceintes ont été enregistrées. L’âge moyen des patientes était de 26 ± 6 ans en 2008, 2009 et 2010. La prévalence globale du VIH était passée de 3,4% en 2008 à 2,9% en 2016 (p = 0,0145). Elle est passée de 1% en 2008 à 0,5% en 2016 et de 3,6% en 2008 à 1,4% en 2016 (p < 0,0001) respectivement chez les 15-19 ans et les 20-24 ans. La prévalence de la syphilis baisse de façon significative de 2008 (1,3%) à 2016 (0,6%), (p < 0,0001). Elle est faible et non associée à l’âge en 2008, alors qu'elle était de 0,2% et 0,4% en 2016 respectivement dans la tranche d’âge de 15-19 ans et de 20-24 ans.

**Conclusion:**

Notre étude documente une prévalence relativement faible de la syphilis et du VIH chez la femme enceinte au Togo, avec une baisse significative chez les adolescentes et les jeunes femmes témoignant de l'efficacité de l'augmentation du dépistage et de la prévention globale des IST, du VIH y compris l'approche traitement antirétroviral comme prévention (TASP, Treatment as prevention), et du programme d’élimination de la syphilis néonatale dans le pays.

## Abstract (en)

**Introduction:**

The aim of our work was to analyse the trends of HIV infection and syphilis among pregnant women in prenatal consultation (PNC) in healthcare facilities in Togo.

**Methods:**

This was an analytical retrospective study, covering the period from 2008 to 2016 and focusing on pregnant women aged 15 to 49 seen in PNC for the first time in maternal and child health services in Togo.

**Results:**

During the study period, 41,536 pregnant women were registered in 2008, 2009, 2010, 2014 and 2016, respectively 8079, 8572, 8430, 7920 and 8535.The mean age of the patients was 26 ± 6 year in 2008, 2009 and 2010. The overall HIV prevalence decreased from 3.4% in 2008 to 2.9% in 2016 (p = 0.0145). It fell from 1% in 2008 to 0.5% in 2016 and from 3.6% in 2008 to 1.4% in 2016 (p < 0.0001) among 15-19 year-old and 20-24 year-old respectively. HIV prevalence in rural areas is two times lower than in urban areas between 2008 and 2016 with a statistically significant difference. The prevalence of syphilis decreased significantly from 2008 (1.3%) to 2016 (0.6%), (p < 0.0001). It is low and not associated with age in 2008; 0.2% and 0.4% in 2016 respectively in the 15 to 19 and 20 to 24 age groups. This prevalence is significantly low between 2008 and 2016 in both urban and rural areas.

**Conclusion:**

Our study documents a relatively low prevalence of syphilis and HIV among pregnant women in Togo, with a significant decrease among adolescents and young women, attesting to the effectiveness of the increased screening and comprehensive prevention of sexually transmitted infections (STIs) and HIV, including the antiretroviral treatment as prevention (TASP) approach, and the neonatal syphilis elimination programme in the country.

## Introduction

Les infections sexuellement transmissibles (IST) et le VIH constituent un enjeu de santé publique depuis plusieurs années dans le monde et surtout dans les pays en voie de développement [3]. Parmi ces IST, le VIH et la syphilis sont associés à une grande morbi-mortalité materno-foetale [7, 8, 20].

En 2019, le nombre de nouvelles infections à VIH était estimé à 1,7 million sur le plan mondial [17]. La prévention, la surveillance épidémiologique et l'amélioration à l'accès aux services de prise en charge des IST et du VIH/Sida sont des composantes importantes dans les programmes de santé publique au niveau mondial et en Afrique [15]. Ainsi, dans la mise en oeuvre des programmes de lutte contre le VIH et le Sida, la connaissance des tendances de prévalence du VIH et des groupes de population affectés par ce fléau aide à mesurer les progrès réalisés et à renforcer les stratégies et approches de prévention. En effet, à la fin des années 1990, l'Organisation mondiale de la Santé (OMS) a recommandé une surveillance sentinelle annuelle notamment chez les femmes enceintes, afin de disposer des informations stratégiques sur la dynamique de l’épidémie du VIH en Afrique [5, 6, 16]. Le but de notre étude était d'analyser les tendances de l'infection à VIH et de la syphilis chez les femmes enceintes en consultation prénatale (CPN) dans les structures de soins du Togo. Les objectifs spécifiques étaient d’étudier ces tendances chez les jeunes et les adolescentes.

## Méthodes

### Organisation de la surveillance sentinelle au Togo

Au Togo, dans le cadre de lutte contre le Sida, le Programme national de Lutte contre le Sida et les Infections sexuellement transmissibles (PNLS-IST) du ministère de la Santé a mis en place une stratégie de surveillance du VIH afin de disposer des informations stratégiques pour orienter la prise de décisions programmatiques. Ainsi depuis 2004, le pays a adopté le protocole de l'OMS de surveillance de seconde génération.

*Population cible.* Femmes enceintes âgées de 15 à 49 ans. Étant donné que la prévalence de l'infection par le VIH dans ce groupe est supposée être inférieure à 10%, une taille minimale de 200 femmes par site suffisait pour obtenir une précision de 5%.

*Critères d'inclusion*. Toutes les femmes enceintes se présentant en CPN pour la première fois pendant la période de la surveillance, acceptant de donner du sang veineux pour le dépistage de la syphilis et du VIH, **et répondant aux critères de sélection (15 à 49 ans) ont** été incluses de façon consécutive jusqu’à l'atteinte de la taille de l’échantillon requise. Les tests utilisés étaient ceux homologués par le ministère de la Santé et répondant au protocole national élaboré par le PNLS-IST.

*Critères de non-inclusion*. Femmes enceintes référées par une autre formation sanitaire, femmes venant en CPN pour la deuxième fois pendant la même période de la surveillance (le carnet de CPN de la femme est le document de contrôle), femmes ayant refusé le prélèvement de sang pour des raisons non précisées dans les rapports. Ces critères ont été adoptés pour éviter les biais et les doublons.

*Sélection des sites sentinelles.* Un site sentinelle est un ensemble de formations sanitaires, c'est-à-dire un centre de Protection maternelle et infantile (PMI), qui accueille les femmes de différents profils sociodémographiques en CPN dans une zone géographique donnée (village, canton, ville ou district) [18].

Les sites ont été sélectionnés selon les critères définis dans le protocole OMS de deuxième génération actualisé et validé par le groupe de référence pour le suivi et l’évaluation. Ainsi depuis 2008, 33 sites (17 sites urbains et 16 sites ruraux) ont été retenus et correspondaient aux centres de santé maternelle et infantile offrant des services du programme de prévention de la transmission du VIH de la mère à l'enfant. La dénomination de site rural a été définie selon les caractéristiques du service national de la statistique et selon les considérations de chaque district sanitaire.

*Aspects éthiques*. Le protocole d'enquête de surveillance a été validé par le comité bioéthique pour la recherche en santé du ministère de la Santé [18], et le consentement éclairé de chaque patiente a été obtenu.

Collecte de données lors de cette étude : Notre étude était rétrospective, consistant à analyser les données et les rapports issus de la surveillance sentinelle menée chez les femmes enceintes entre 2008 et 2016. Il s'agissait d'une étude à la fois descriptive portant sur les caractéristiques démographiques, et surtout analytique des tendances évolutives des prévalences du VIH et de la syphilis.

*Analyse statistique.* Elle a été faite grâce au logiciel Rstudio version 3.6.3. Les variables quantitatives ont été décrites en moyennes +/- écart type. Les variables qualitatives ont été décrites en effectifs et pourcentages; et ont été comparées grâce aux tests de chi2 ou Fisher avec comme seuil de significativité une valeur de p inférieure à 0,05.

## Résultats

Le nombre de femmes enceintes dépistées en 2008, 2009, 2010, 2014 et 2016 étaient respectivement de 8 079, 8 572, 8 430, 7 920 et 8 535 avec un âge moyen de 26 ± 6 ans en 2008 (Tableau I).

**Tableau I:** Prévalence de l'infection à VIH chez les femmes enceintes en consultation prénatale Prevalence of HIV infection among pregnant women attending antenatal clinics

	2008	2009	2010	2014	2016	p
**Nombre de femmes testées**	8 079	8 572	8 430	7 920	8 535	
**Âge moyen (ans)**	26 ± 6	26 ± 6	26 ± 6	25,8	2 6,7	
**Effectifs dans les sites urbains**	4 400	4 966	4 550	4 358	4 496	
**Effectifs dans les sites ruraux**	3 679	3 606	3 880	3 562	4 039	
**Prévalence globale (%) (15-49 ans)**	3,4	3,9	3,5	3,3	2,9	0,0145
**Prévalence dans les sites urbains (%)**	4,4	4,6	4,4	4,4	3,4	0,001
**Prévalence dans les sites ruraux (%)**	2,3	3	2,5	1,9	2,3	0,0002
**Prévalence selon les tranches d’âge**
**15-19 ans**	1	2,1	1,5	0,9	0,5	< 0,0001
**20-24 ans**	3,6	3,6	2,6	1,9	1,4	< 0,0001

### Évolution de la prévalence de l'infection VIH

**Figure 1:**
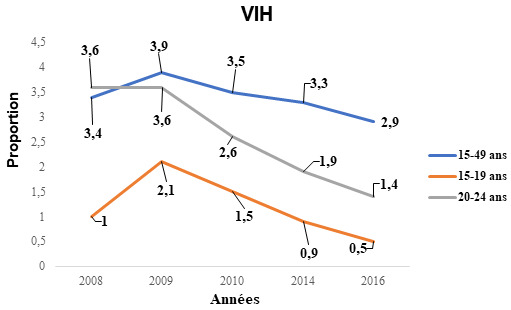
Évolution de la prévalence du VIH chez les femmes enceintes selon les tranches d’âge Trends in HIV prevalence among pregnant women by age group

La prévalence globale du VIH chez les femmes de 15-49 ans était passée de 3,4% en 2008 à 2,9% en 2016 (p = 0,0145). En considérant les tranches d’âges stratégiques que sont celles de 15-19 ans et 20-24 ans, les tendances montrent une baisse entre 2008 et 2016. Elle est passée de 1% en 2008 à 0,5% en 2016 dans la tranche d’âge de 15-19 ans (p < 0,0001); dans la tranche d’âge de 20-24 ans, elle est passée de 3,6% en 2008 à 1,4% en 2016 (p < 0,0001) (Fig. 1). La prévalence du VIH en milieu rural est 2 fois plus faible qu'en milieu urbain entre 2008 et 2016 avec une différence statistiquement significative. On note aussi une baisse dans les sites urbains : 4,4% en 2008 *versus* 3,4% en 2016 (p = 0,0002), (Tableau I).

### Évolution de la prévalence de la syphilis

**Figure 2:**
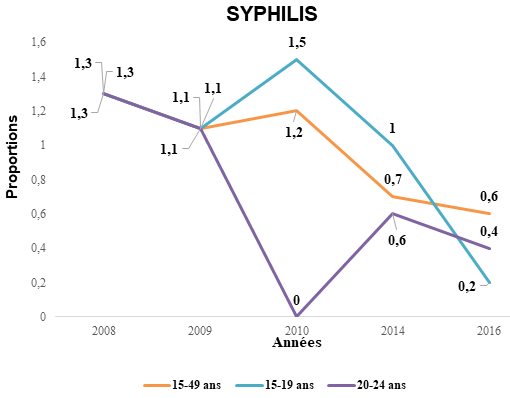
Évolution de la prévalence de la syphilis chez les femmes enceintes selon les tranches d’âge (en 2008 la prévalence de syphilis était identique dans les trois tranches d’âge, soit de 1,3%, ainsi qu'en 2009 avec une prévalence de 1,1%) Trends in syphilis prevalence among pregnant women by age group (in 2008 the prevalence of syphilis was identical in the three age groups, i.e. 1.3%, as well as in 2009 with a prevalence of 1.1%)

La prévalence globale de la syphilis baisse de façon significative de 2008 (1,3%) à 2016 (0,6%), (p < 0,0001). Cette tendance en fonction des tranches d’âges montre des variations entre 2008 et 2016. Elle est faible et non associée à l’âge en 2008 : alors qu'elle était de 0,2% et 0,4% en 2016 respectivement dans la tranche d’âge de 15-19 ans et de 20-24 ans (Fig. 2). Cette prévalence est significativement basse entre 2008 et 2016, aussi bien en milieu urbain que rural (Tableau II).

**Tableau II:** Prévalence de la syphilis chez les femmes enceintes en consultation prénatale Prevalence of syphilis among pregnant women attending antenatal clinics

	2008	2009	2010	2014	2016	p
**Nombre de femmes testées**	8 079	8 572	8 430	7 920	8 535	
**Âge moyen (ans)**	26 ± 6	26 ± 6	26 ± 6	25,8	26,7	
**Effectifs dans les sites urbains**	4 400	4 966	4 550	4 358	4 496	
**Effectifs dans les sites ruraux**	3 679	3 606	3 880	3 562	4 039	
**Prévalence globale (%) (15-49 ans)**	1,3	1,1	1,2	0,7	0,6	< 0,0001
**Prévalence dans les sites urbains**	1,3	1,1	0,9	0,7	0,5	< 0,0001
**Prévalence dans les sites ruraux (%)**	1,3	1,1	1,5	0,8	0,7	< 0,0001
**Prévalence selon les tranches d’âge**
**15-19 ans**	1,3	1,1	1,5	1	0,2	< 0,0001
**20-24 ans**	1,3	1,1	-	0,6	0,4	< 0,0001

## Discussion

La baisse significative de la prévalence du VIH est bien illustrée chez les adolescentes de 15-19 ans et les jeunes femmes de 20-24 ans, tranches d’âge où apparaissaient les nouvelles infections. Cette tendance est corroborée par la tendance générale de l'infection par le VIH dans le pays qui a connu une inversion significative des nouvelles infections à partir de 2010. En effet, entre 2000 et 2015, le pays a enregistré une baisse de nouvelle infection de 60% [2]. En dehors des plans stratégiques nationaux de lutte contre le Sida, le pays a mis en place depuis 2004 un programme dynamique de prévention de la transmission du VIH de la mère à l'enfant qui est monté progressivement en puissance en termes de couverture géographique et de cible.

Les populations cibles vulnérables en Afrique restent les femmes et notamment les adolescentes. D'où l'importance de renforcer l'intégration d'offres de services de VIH et de la santé sexuelle et d'offrir un paquet de services adaptés aux jeunes et aux adolescents.

En Afrique subsaharienne, du fait de la transmission du VIH par voie majoritairement hétérosexuelle, la surveillance sentinelle chez les femmes enceintes donne des informations stratégiques sur la dynamique de l’épidémie du VIH au sein de ce groupe assez représentatif de la population générale. La surveillance sentinelle complète les études populationnelles de type enquête démographique et santé qui, compte tenu de leur coût, ne peuvent se faire régulièrement [1].

Les tendances évolutives de la prévalence du VIH au Togo ont été observées à divers degrés dans d'autres pays comme le Congo [14], la Tanzanie [10, 12] et confirment la dynamique baissière de la prévalence du VIH dans la population générale en Afrique subsaharienne.

La prévalence globale du VIH rapportée dans notre étude chez les 15-49 ans en 2016 (2,9%) est supérieure à celle rapportée au Bénin en 2018 (2%) [19]. Cette prévalence est en revanche inférieure à celles retrouvées en Afrique centrale, principalement en République du Congo (3,6%) [14], en Centrafrique (4,7%) [4], en Tanzanie (5,6%) [12] et en Afrique de l'Est et notamment en Éthiopie (10,33%) [13]. Dans notre étude, la prévalence du VIH en milieu rural est 2 fois plus faible qu'en milieu urbain entre 2008 et 2016 avec une différence statistiquement significative. Par rapport à l’âge, les tendances montrent une baisse entre 2008 et 2016. Ces variations peuvent résulter des différences de culture, de statut socio-économique, du risque de comportement sexuel, de prévention et des mesures de contrôle, des facteurs de risque, du niveau de sensibilisation et des méthodes utilisées pour le diagnostic [13].

Les résultats de notre étude montrent un faible taux de positivité dans les tranches de 15 à 19 ans, passant de 1% (2008) à 0,5% (2016) *versus* 2,8% en République du Congo [14]. Nos résultats sont inférieurs à ceux de Manyahi *et al.* en Tanzanie avec respectivement 3,9% pour les tranches de 15-24 ans, 7,3% pour les 25-34 ans et 7% chez les plus de 35 ans [12]. Niama *et al.* au Congo ont trouvé une plus grande séropositivité chez les plus de 25 ans (4,4%) [14]. Ces faibles taux chez les jeunes de 15 à 19 ans et 20 à 24 ans dans notre étude pourraient s'expliquer par le fait que les mesures de prévention pour réduire ce fléau sont respectées dans ce groupe.

Quant à la syphilis, il y a eu une baisse significative dans notre étude par rapport aux autres pays analysés aussi bien chez les jeunes et adolescentes que chez les femmes plus âgées (Fig. 2), documentant ainsi la faible morbidité de cette affection au Togo après les programmes dynamiques de prévention et de prise en charge des IST classiques d'une part, et la prévention combinée mise en place pour le VIH d'autre part. Ceux-ci, mis en oeuvre dans le pays depuis plusieurs années, étaient associés à l’élaboration d'un plan d’élimination de la syphilis néonatale [18].

En Afrique subsaharienne, la prévalence de la syphilis parmi les femmes enceintes a été estimée entre 0,6% au Sénégal et 14% en Guinée équatoriale [9]. Dans notre étude, on note une absence de différence en fonction de l’âge et une évolution globale à la baisse quels que soient les sous-groupes, notamment de 0,2% et 0,4% en 2016 respectivement dans les tranches de 15 à 19 ans et de 20 à 24 ans. Des prévalences plus élevées ont été rapportées par Niama *et al.* au Congo (3,9%) [14] et Gamba *et al.* en Centrafrique (7,6%) [4]. La séroprévalence de la syphilis en Éthiopie était de 3,7%, et de 3% à Madagascar [13].

Dans notre étude, la prévalence de la syphilis était significativement basse entre 2008 et 2016 aussi bien en milieu urbain que rural. En Tanzanie, elle était plus élevée chez les femmes enceintes vivant en milieux ruraux (16,0%) par rapport à celles des milieux urbains (7,0%) [10]. Une étude menée en Zambie a trouvé une prévalence de la syphilis qui a chuté davantage chez les femmes enceintes résidant en milieu urbain (9,8% à 2,8%) par rapport à celles résidant en milieu rural (7,5% à 3,2%), [11]. La surveillance sentinelle a permis à certains pays comme le Cameroun de noter une résurgence de la syphilis chez les femmes enceintes [9].

Certes, la méthodologie utilisée dans notre étude aussi bien pour le VIH que la syphilis (période, population cible, stratégie de recrutement, compliance, technique d'analyse, représentativité des femmes) n'est pas la même pour les autres études. Cependant, la comparaison des résultats obtenus nous permet de tirer des conclusions pour mener des actions à l'endroit de ces populations.

## Conclusion

Notre étude documente une prévalence relativement faible de la syphilis et du VIH chez la femme enceinte au Togo, avec une baisse significative chez les adolescentes et les jeunes femmes. Les tendances montraient une diminution significative avec le temps suite à l'augmentation du dépistage, la prévention globale des IST, du VIH y compris l'approche par traitement antirétroviral comme prévention (TASP), et le programme d’élimination de la syphilis néonatale dans le pays. Néanmoins, des efforts substantiels sont encore nécessaires à travers les plans d’élimination de la transmission du VIH et de la syphilis de la mère à l'enfant afin de consolider les résultats obtenus dans ce groupe de population et par extension dans la population générale. Ces études de surveillance ciblant certaines populations (femmes enceintes, militaires, populations clés) sont utiles, car elles fournissent des données sur la dynamique de l’épidémie dans le groupe correspondant et sur l'impact des interventions et stratégies menées.

## Remerciements

Les auteurs voudraient remercier le ministère de la Santé, de l'hygiène publique et de l'accès universel, le Programme national de Lutte contre le Sida (PNLS) et le Conseil national de Lutte contre le Sida (CNLS) d'avoir financé les différentes enquêtes de surveillance sentinelle et de nous avoir autorisés à utiliser les données disponibles pour la réalisation de cette étude.

## Liens D'intérêts

Les auteurs ne déclarent aucun lien d'intérêt.

## Contribution des Auteurs

AS, AD, KK et PP : conception de l’étude, analyse, interprétation, rédaction du manuscrit.

ASA, AL, BS : participation à la conception de l’étude, l'analyse et l'interprétation des données et rédaction du manuscrit.

JNT : implication dans l'analyse et l'interprétation, écriture et finalisation du manuscrit.

Tous les auteurs étaient responsables de la gestion scientifique globale de l’étude, de l'analyse et de l'interprétation. Enfin, tous les auteurs ont lu et approuvé le manuscrit final à soumettre pour publication.
